# Children’s Environmental Health Indicators in Context of the Sustainable Development Goals for Small Island Developing States

**DOI:** 10.3390/ijerph15071404

**Published:** 2018-07-03

**Authors:** Eun Mi Jung, Paul Jagals, Claire Brereton, Peter D. Sly, Rokho Kim, Eun Mee Kim, Eun Hee Ha

**Affiliations:** 1 Department of Occupational and Environmental Medicine, College of Medicine, Ewha Womans University, 07985 Seoul, Korea; mejung@ewhain.net; 2 Ewha Global Health Institute for Girls, College of Medicine, Ewha Womans University, 07985 Seoul, Korea; 3 The University of Queensland, Child Health Research Centre, 4101 Brisbane, Australia; p.jagals@uq.edu.au (P.J.); claire.brereton@uq.edu.au (C.B.); p.sly@uq.edu.au (P.D.S.); 4 School of Public Health and Primary Care, Fiji National University, Suva, Fiji; 5 Health and the Environment, WHO Regional Office for the Western Pacific, 1000 Manilla, Philippines; kimr@who.int; 6 Department of International Studies, Graduate School of International Studies, Ewha Womans University, 03760 Seoul, Korea

**Keywords:** sustainable development goals, SDG, sustainable development impacts, children’s environmental health indicators, CEHI, small island developing states, SIDS, pacific island small states, PSS

## Abstract

The unique environmental vulnerability of small island developing states (SIDS) is likely to impact negatively on children’s health. Children’s environmental health indicators (CEHI) are standardized measures that can be used to assess the environmental exposures and their resulting health outcomes in children. This study sought to utilize the United Nations (UN) global Sustainable Development Goals (SDGs), with their associated targets and indicators, as a framework for a CEHI proposal for SIDS. Exposure-side indicators were taken from key themes from the 2012 Rio+20 UN Conference on Sustainable Development, and health-side indicators were selected based on the most significant contributors to the burden of disease in children. The multiple-exposures–multiple-effect (MEME) framework was then used to show the relationships between environmental exposures and children’s health outcomes. The framework was populated with available data from the World Bank’s DataBank. Whilst there was some data available at a population level, major gaps in both exposure-side and health-side indicators were revealed. In order to progress children’s environmental health in SIDS, a further piece of work is required to propose a fully prioritized set of exposure-side and health-side CEHIs; based on, but not exclusively linked to, the SDGs.

## 1. Introduction

The key to a peaceful, prosperous, and sustainable world is healthy, safe, and empowered children [1]. Because children are particularly vulnerable to environmental risks to their sustained and healthy development [2], creating a safe and healthy environment is one of the essential elements for improving child survival, helping children to thrive, and achieving health equity [3]. In fact, studies have identified a significant link between the burden of disease and environmental risks [4,5]. Global estimates suggest that 26% of all childhood deaths, and a quarter of the disease burden in children aged under five, could be prevented by removing modifiable environmental risks [5]. The disease burden linked to the environment also tends to be higher in low- and middle-income countries (LMICs) [6]. However, relatively little attention has been paid to the role of the environment on children’s health [7].

The United Nations (UN) Sustainable Development Goals (SDGs) [8] encourages an inter-sectoral approach to resolve the shortcomings of health goals in the Millennium Development Goals and the emerging environmental health challenges. Furthermore, the SDGs provide strong guidance for governments around the world, to monitor and assess their progress (or lack thereof) towards creating healthier environments for children [3]. The goals target several environmental health risk factors and provide the indicators to measure their status and progress towards reducing these risks [9].

Disaggregated data collected through scientific, robust, and comprehensible indicators will play a crucial role in identifying and reaching children who have high risks of being exposed to environmental risks [1]. However, many LMICs will find it difficult to routinely collect such robust data because of the lack of suitable monitoring and surveillance tools integrated into systems of information flow and decision-making. Small island developing states (SIDS) are LMICs in a particularly vulnerable position, with high environmental vulnerability and low economic growth [10]. SIDS are examples of countries who will face significant challenges in measuring their progress toward creating the environments in which children could “survive, thrive, and transform” [3].

Adopting indicators specifically for children is crucial for assessing, monitoring, and evaluating children’s environmental health status [11]. Furthermore, the data derived from the indicators can be used to establish policies that help improve children’s health and well-being and reduce environmental risks. SIDS, in common with other countries, have a significant political focus on progress towards the SDGs, and a number of the governments and international organizations routinely monitor and report the SDG indicators. Utilizing existing SDG indicators would have a number of benefits, including reduced burden of information collection and harmonization of measurements between countries.

This paper explores the extent to which the indicators related to environmental health risks and impacts under the SDG targets would inform policy makers of the status of children’s environmental health. It is the premise of this paper that the SDG targets and their indicators will need to be supplemented and adapted to meet the unique requirements for measuring environmental health risks and impacts on children. The objective of this study was two-fold: (1) to illustrate how the selected SDG indicators can be employed for tracking children’s environmental health status in SIDS and (2) to address the need for more child-specific and age disaggregated data to effectively monitor and evaluate children’s environmental health in SIDS by presenting openly accessible data from the World Bank.

## 2. Materials and Methods

**Figure 1:**
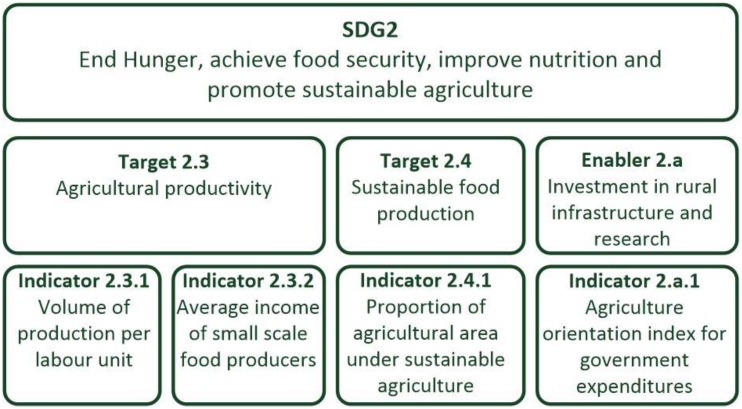
Sample Sustainable Development Goals (SDG) hierarchy with numbering.

We reviewed the global indicator framework for the SDGs [12] to select appropriate indicators that can be used to express the relationship between environmental exposures and health outcomes for children. The SDGs were adopted in 2015 by the United Nations, succeeding the expiring Millennium Development Goals (MDGs). They were intended to complete the ‘unfinished business’ of the MDGs and to extend the scope, addressing the global implications of development for all states and covering broad agendas such as biodiversity, sustainability, and pollution [13]. The 17 SDGs are broad goals, broken down into more detailed targets, with progress towards each target measured by a number of indicators (Figure 1). Both targets and indicators may contain a range of topics and measures. A set of CEHI based on SDGs may therefore need to create a further subset of the indicators, with disaggregation as required to get child-specific data.

When selecting exposure-side indicators, we first set the strategic themes in relation to the leading topics of the 2012 Rio+20 UN Conference on Sustainable Development, which were food; water, sanitation, and hygiene (WASH); energy; cities; and climate change [14]. We modified the Rio+20 theme of ‘cities’ to ‘cities and human settlements’ to correspond with SDG 11, as well as to ensure that the many settlements outside cities in SIDS were considered. Because geographical characteristics and the development status of SIDS can make their cities and human settlements particularly vulnerable to climate change [15], we linked climate change to cities and human settlements and concentrated on showing the multiple effects of climate change and unsustainable settlements on children’s health. In addition, water contamination was identified as one of the major problems in waste management in SIDS [16], waste management was incorporated as part of WASH.

For health-side indicators, we particularly focused on indicators related to the most significant contributors to the burden of disease in children of LMICs [9]. Therefore, we selected respiratory diseases, diarrheal diseases, malaria, and malnutrition as our main health outcomes. We also selected a health-side indicator addressing disasters as one of our main health-side indicators, because SIDS are prone to natural disasters, often associated with extreme weather events and climate change [17].

To construct children’s environmental health indicators (CEHI) for SIDS, the multiple-exposures–multiple-effect (MEME) framework, which was specifically developed for improving, monitoring, and evaluation of children’s environmental health [2], was populated with the selected SDG indicators and targets. The MEME framework was chosen because it allows one to describe the complex relationship between exposures and health outcomes with integrating socio-economic contexts and policy actions into the framework. Within each strategic theme, exposure-side indicators and context indicators were selected and health-side indicators were placed alongside the related exposure or context. Health outcomes were set by using SDG targets. Related SDGs were stated separately to show the general objectives of the whole CEHI process. Because some measures that are required to describe the context of the relationship between environmental exposures and health outcomes were not readily available in the indicators list, we also reviewed indicators from the MEME framework and included its indicator in the list.

With robust data provided, the SDG indicators integrated into the MEME framework would enable monitoring and evaluation of children’s environmental health. We searched the World Bank’s DataBank [18] for the data from 2010 to 2017 for data availability. We used the DataBank as a sole source of data because other international organizations’ methods of country classifications were different from the World Bank’s. The World Bank classifies small states into three groups: Caribbean small states (CSS), Pacific Island small states (PSS), and other small states [19]. Data for PSS were used as the closest equivalent for SIDS because the PSS group contains the largest number of LMICs and exclusively covers island states: nine states included in the PSS were Fiji, Kiribati, Marshall Islands, Federated States of Micronesia, Samoa, Solomon Islands, Tonga, Tuvalu, and Vanuatu [20]. CSS contains seven LMICs, including Belize, Dominica, Grenada, Guyana, Jamaica, Saint Lucia, and Saint Vincent and the Grenadines [20].

## 3. Results

Table 1 shows indicators from both the SDGs and the MEME frameworks. The exposure-side and health-side indicators, SDG targets, and goals were classified according to the strategic themes and placed in the MEME framework. Eleven exposure-side or context indicators related to food, WASH, energy, and cities and human settlements associated with climate change were selected from the global indicator framework and the MEME framework. For instance, poverty was viewed as a context indicator for food as poverty can create a vicious cycle that involves malnutrition [21]. Indicators on water, sanitation, hygiene, and waste management were matched to WASH as stated above.

Considering that energy is related to access to clean fuel and use of renewable energy sources [22], energy was associated with indoor and outdoor air pollution. CO_2_ emissions were placed as a context indicator for climate change because CO_2_ emissions are generally regarded as one of the main causes of climate change [23]. In addition, a MEME indicator on assessing the number of children with an increased risk of malaria transmission was used as a exposure-side indicator for climate change because climate change has been suspected to increase malaria-transmissible areas [24].

Health outcomes were composed of seven SDG health-side indicators on malnutrition, diarrheal diseases, respiratory diseases, malaria, and natural disasters. Thus, these indicators were mapped to food, WASH, energy, and climate change, respectively. Food was related to malnutrition because of the link between inadequate food intake and malnutrition. WASH was linked to the WASH mortality rate instead of mortality as a result of diarrheal disease, because there is no health-side indicator addressing diarrheal diseases in the global indicator framework for the SDGs.

For energy, because the use of biomass fuel is a major source of household air pollution [25], access to clean fuels and technologies for cooking and heating can reduce the mortality rate attributed to household air pollution. Fossil fuel combustion can generate a number of air pollutants including sulfur oxides, nitrogen oxides, and particulate matter [26], all of which can cause outdoor air pollution. It has been inferred that these air pollutants could result in pulmonary diseases [27]. Therefore, indicator 3.4.1 was used as a health-side indicator for energy because it includes chronic respiratory disease as health outcomes of interest.

Although there is a wide variety of effects of climate change on children’s health [28], climate change was specifically linked with malaria and natural disasters because of their relevance to SIDS. Furthermore, as climate change has been suspected to be linked to increased risks of mosquito-borne diseases [29], malaria was selected as a health outcome for climate change. As the frequencies of extreme events, including droughts, floods, and extreme temperatures, can be affected by climate change [30], climate change is thought to be a potential contributor to natural disasters.

Actions consisted of nine SDG targets corresponding to outcomes. Targets tackling health outcomes tend to involve multiple health outcomes in one target. Thus, health outcome targets were included if those targets state the health outcome of interest. For instance, WASH has four targets, two of which are health outcome targets. Because target 3.3 states water-borne diseases and target 3.4 states mortality due to water contamination, presumably related to diarrheal diseases, targets 3.3 and 3.4 were used as action indicators for WASH. Similarly, target 3.4 could be incorporated as an action indicator for energy because non-communicable diseases encompass chronic respiratory diseases. Because target 3.3 has malaria as one of the health outcomes of interests, it also links to climate change. The target on natural disasters was also regarded as an action indicator for climate change. Targets on poverty reduction and improving food security were employed for food.

Seven SDGs that indicate general objectives of each CEHI process are shown. SDG 3, “Ensure healthy lives and promote well-being for all at all ages [12]”, was included as a common goal and each strategic theme has corresponding goals.

Table 2 provides data variables and data availability for the selected exposures. One indicator could have a number of data variables in the DataBank. Labels of data variables presented here are identical to those in the DataBank. Discrepancies between, as well as within, strategic domains were observed. Accessibility to data on climate change was relatively high. Data on WASH and energy were only available at a basic level, such as percentage of people using basic drinking water services and mean annual concentration of PM_2.5_. An advanced level of data, including people having access to safely managed drinking water or people having exposure to PM_2.5_ concentration exceeding WHO interim target-3 level, was not readily accessible. For WASH, indicators on waste management and water quality did not even have data variables specified in the DataBank. Data for poverty were not found in the Databank. Even though some of the exposure-side indicators were disaggregated by location of residence, none of the data were child specific.

Table 3 shows data variables and data availaiblty for the selected health outcomes. Health data were rarely available for PSS. Data have been collected for only two health outcomes; namely prevalence of undernourishment and mortality from cardiovascular disease, cancer, diabetes, or chronic respiratory disease between the exact ages 30 and 70. However, data were not child-specific. For other indicators, data were either not available or data variables did not exist in the Databank. Health-side indicators on food were the only indicators that are child-specific, but no data have been collected.

## 4. Discussion

The SDGs and MEME indicators were formulated around the MEME framework to illustrate the relationship between the selected environmental risks and health consequences in children. The four strategic themes, namely, food, WASH, energy, and climate change, associated with cities and human settlements were selected because of their significance in the SDGs and relevance to situations in SIDS. For instance, food insecurity has been a major concern in SIDS [32], and SIDS heavily rely on combustion of fossil fuels [33] to generate energy. Depletion of fresh water, lack of proper sanitation facilities, and inappropriate treatment of waste water have been environmental threats to SIDS [34], and SIDS are vulnerable to the imminent impacts of climate change [35].

The strategic themes were linked to the leading causes of childhood mortality [6,9], preventable through improving environmental conditions. The limitation of this approach was that we linked a single environmental exposure to a specific health outcome, but there are many cases where multiple exposures can cause multiple health outcomes in a complex relationship. However, use of the MEME framework can reduce the bias because it allows a more systematic approach to the complex relationship between exposures and health outcomes.

The indicators in the framework can be used to generate scientific data for SIDS. In addition, because of their relevance to the SDGs, the indicators can inform policy makers of the glimpse of the current environmental health status. To determine the data availability and simulate future usage, data from the World Bank’s DataBank were presented.

PSS were used as a probe for SIDS because PSS encompassed the largest number of LMICs and exclusively contained island states. CSS include Belize and Suriname, which are not island countries [36]. Because the common characteristics can stem from the geographical peculiarity of islands [37], using PSS as an example was plausible. The shortcoming that was found in the scrutiny of data was that because the SDG indicators were developed to be used at country level to feed into a global level perspective, they are not ideally fitted for assessing children’s environmental health status for SIDS. Therefore, SIDS should develop complementary/supplementary indicators that meet their national needs.

In addition, none of the exposure data were disaggregated by age. Similarly, the health-side indicators in the SDGs are mostly not child-specific. Even though the guidelines for SDG indicator measurement include data disaggregation into age bands where possible [38], it is unclear whether source data from SIDS contain the detail required to achieve this. We know that children are disproportionately affected by environmental exposures because of their unique exposure pathways and their growing bodies and systems [39], making it especially important to collect disaggregated data by age group to track progress in children’s health.

Furthermore, data robustness could also be enhanced through collection and summarization of age metadata. Analysis of age-disaggregated data would then enable identification of susceptible age groups and analysis of health status gaps between age groups. In addition, this information would help prioritize the allocation of limited resources in SIDS. Thus, it is inferred that disaggregating these indicators by age will be required before adopting the indicators for assessing children’s environmental health status in SIDS.

Collection of data on both environmental risks and the related health outcomes provides a basis for identifying the effects of environmental interventions on health outcomes. Data availability was appraised for each strategic theme and was found to be mostly unavailable—especially in health outcomes. Only 2 out of 18 data variables have data collected in health outcomes.

In addition to the lack of data availability, there was a tendency for a number of health outcomes to be included in one health-side target and separate data variables were not provided in the data list. For instance, health-side target 3.3; “By 2030, end the epidemics of Acquired Immune Deficiency Syndrome (AIDS), tuberculosis, malaria, and neglected tropical diseases and combat hepatitis, water-borne diseases, and other communicable diseases [12]”, did not have a disaggregated data variable for water-borne diseases in the Databank. If a data variable is missing, the corresponding data would not be accessible either.

Similarly, exposure data were not appropriately elaborated in the data. For instance, energy only focused on access to clean fuel, while improved stove could be more feasible in LMICs settings. Hence, it is crucial to specify a complete set of indicators in the data variables to collect robust data and monitor children’s environmental health status for SIDS.

## 5. Conclusions

This study proposes a coherent set of the most important CEHIs for SIDS, using the MEME framework to show the linkages between exposure-side indicators and health-side indicators. The SDG goals, targets, and indicators, supplemented by indicators from the MEME children’s environmental health framework, provide a common reference point. There are many advantages to developing a set of CEHIs that are based on SDG targets and indicators. SIDS are required to regularly report progress on SDGs so a proposal for collection of data , which contributes to this reporting, will align with existing focus areas.

Whilst most of the SDG indicators are not child-specific, the guidelines for data collection request disaggregation into age and gender groups. This disaggregated data is unavailable in the World Bank DataBank, but some may be available from other sources. Not all of the SDG indicators are ideally suited for assessing children’s environmental health, and SIDS should develop complementary/supplementary indicators for more specific exposure-side and health-side indicators.

Setting up a data collection process and system based on a suitable set of indicators will provide evidence that will help SIDS to identify and target their most important children’s environmental health issues.

## Figures and Tables

**Table 1:** Children’s environmental health indicators designed for small island developing states (SIDS) *.

**A. Strategic Theme: Food**	
**Contexts/Exposures**	**Related SDGs**
1.2.1 Proportion of population living below the national poverty line, by sex and age	Goal 1. End poverty in all its forms everywhere Goal 2. End hunger, achieve food security and improved nutrition, and promote sustainable agriculture Goal 3. Ensure healthy lives and promote well-being for all at all ages
Health outcomes	
2.2.1 Prevalence of stunting (height for age <−2 standard deviation from the median of the World Health Organization (WHO) Child Growth Standards among children under five years of age	
2.2.2 Prevalence of malnutrition (weight for height >+2 or <−2 standard deviation from the median of the WHO Child Growth Standards among children under five years of age, by type (wasting and overweight)	
Actions	
1.2 By 2030, reduce at least by half the proportion of men, women, and children of all ages living in poverty in all its dimensions according to national definitions	
2.1 By 2030, end hunger and ensure access by all people, in particular the poor and people in vulnerable situations, including infants, to safe, nutritious, and sufficient food all year round	
**B. Strategic theme: Water, sanitation, and hygiene (WASH)**	
**Contexts/Exposures**	**Related SDGs**
6.1.1 Proportion of population using safely managed drinking water services	Goal 3. Ensure healthy lives and promote well-being for all at all ages Goal 6. Ensure availability and sustainable management of water and sanitation for all
6.2.1 Proportion of population using safely managed sanitation services, including a hand-washing facility with soap and water	
6.3.1 Proportion of wastewater safely treated	
6.3.2 Proportion of bodies of water with good ambient water quality	
11.6.1 Proportion of urban solid waste regularly collected and with adequate final discharge out of total urban solid waste generated, by cities	
12.4.2 Hazardous waste generated per capita and proportion of hazardous waste treated, by type of treatment	
Health outcomes	
3.9.2 Mortality rate attributed to unsafe water, unsafe sanitation, and lack of hygiene (exposure to unsafe Water, Sanitation, and Hygiene for All (WASH) services)	
Actions	
3.3 By 2030, end the epidemics of AIDS, tuberculosis, malaria and neglected tropical diseases and combat hepatitis, water-borne diseases and other communicable diseases	
3.9 By 2030, substantially reduce the number of deaths and illnesses from hazardous chemicals and air, water, and soil pollution and contamination	
**C. Strategic theme: Energy**	
**Contexts/Exposures**	**Related SDGs**
7.1.2 Proportion of population with primary reliance on clean fuels and technology	Goal 3. Ensure healthy lives and promote well-being for all at all ages Goal 7. Ensure access to affordable, reliable, sustainable, and modern energy for all
11.6.2 Annual mean levels of fine particulate matter (e.g., PM_2.5_ and PM_10_) in cities (population weighted)	
Health outcomes	
3.4.1 Mortality rate attributed to cardiovascular disease, cancer, diabetes, or chronic respiratory disease	
3.9.1 Mortality rate attributed to household and ambient air pollution	
Actions	
3.4 By 2030, reduce premature mortality from non-communicable diseases by one-third through prevention and treatment and promote mental health and well-being	
3.9 By 2030, substantially reduce the number of deaths and illnesses from hazardous chemicals and air, water, and soil pollution and contamination	
7.1 By 2030, ensure universal access to affordable, reliable, and modern energy services	
**D. Strategic theme: Cities and human settlements - Climate change**	
**Contexts/Exposures**	**Related SDGs**
9.4.1 CO_2_ emission per unit of value added	Goal 3. Ensure healthy lives and promote well-being for all at all ages Goal 11. Make cities and human settlements inclusive, safe, resilient, and sustainable Goal 13. Take urgent action to combat climate change and its impacts
11.1.1 Proportion of urban population living in slums, informal settlements, or inadequate housing	
Children aged 0–14 years living in areas endemic for insect-borne diseases [2]	
Health outcomes	
3.3.3 Malaria incidence per 1000 population	
11.5.1 Number of deaths, missing persons, and directly affected persons attributed to disasters per 100,000 population	
Actions	
3.3 By 2030, end the epidemics of malaria and neglected tropical diseases and combat water-borne diseases and other communicable diseases	
11.5 By 2030, significantly reduce the number of deaths and the number of people affected and substantially decrease the direct economic losses relative to global gross domestic product caused by disasters, including water-related disasters, with a focus on protecting the poor and people in vulnerable situations	

Sources: Briggs, 2003 [2]; United Nations General Assembly, 2014 [31]. * Context/exposure/health outcome/action numbering taken from Sustainable Development Goals (SDG) target and indicator numbering as described in Figure 1. Abbreviations: AIDS, Acquired Immune Deficiency Syndrome; PM_10_, particulate matter less than or equal to 10 μm in diameter; PM_2.5_, particulate matter less than or equal to 2.5 μm in diameter.

**Table 2:** Data on the selected exposures in Pacific Island small states (PSS).

Strategic Theme	Exposure-Side Data Variables	Data Availability
Food	1.2.1 Poverty headcount ratio at national poverty lines (% of population; total, rural, and urban)	Data not available
Water, sanitation, and hygiene (WASH)	6.1.1 People using at least basic drinking water services (% of population; total, rural, and urban)	As for population
	6.2.1 People practicing open defecation (% of population; total, rural, and urban)	
	6.2.1 People using at least basic sanitation services (% of population; total, rural, and urban)	
	6.1.1 People using safely managed drinking water services (% of population; total, rural, and urban)	Data not available
	6.2.1 People using safely managed sanitation services (% of population; total, rural, and urban)	
	6.2.1 People with basic handwashing facilities including soap and water (% of population; total, rural, and urban)	
	6.3.1 Proportion of wastewater safely treated	No data variable
	6.3.2 Proportion of bodies of water with good ambient water quality	
	11.6.1 Proportion of urban solid waste regularly collected and with adequate final discharge out of total urban solid waste generated, by cities	
	12.4.2 Hazardous waste generated per capita and proportion of hazardous waste treated, by type of treatment	
Energy	7.1.2 Access to clean fuels and technologies for cooking (% of population)	As for population
	7.1.2 Access to electricity (% of population; total, rural, and urban)	
	11.6.2 PM_2.5_ air pollution, mean annual exposure (micrograms per cubic meter)	
	11.6.2 PM_2.5_ air pollution, population exposed to levels exceeding WHO guideline value (% of total)	
	11.6.2 PM_2.5_ air pollution, population exposed to levels exceeding WHO Interim Target-1 value (% of total)	Data not available
	11.6.2 PM_2.5_ air pollution, population exposed to levels exceeding WHO Interim Target-2 value (% of total)	
	11.6.2 PM_2.5_ air pollution, population exposed to levels exceeding WHO Interim Target-3 value (% of total)	
Cities and human settlements: climate change	9.4.1 CO_2_ emission per unit of value added (kg per 2010 US$ of GDP)	As for population
	9.4.1 CO_2_ emission per unit of value added (kg per 2011 PPP $ of GDP)	
	9.4.1 CO_2_ emission per unit of value added (kg per PPP $ of GDP)	
	9.4.1 CO_2_ emission per unit of value added (metric tons per capita)	
	Children aged 0–14 years living in areas endemic for insect-borne diseases	Not applicable

(Sources: DataBank from the World Bank Group [18] and Briggs, 2003 [2]). Abbreviations: PM_2.5_, particulate matter less than or equal to 2.5 μm in diameter; WHO, World Health Organization; US$, United States Dollar; PPP, purchasing power parity; GDP, gross domestic product.

**Table 3:** Data on the selected health outcomes in PSS.

Strategic Themes	Health-Side Data Variables	Data Availability
Food	2.2.1 Prevalence of undernourishment (% of population)	As for population
	2.2.1 Prevalence of severe wasting, weight for height (% of children under five; total, female, and male)	Data not available
	2.2.1 Prevalence of stunting, weight for height (% of children under five; total, female, and male)	
	2.2.1 Prevalence of underweight, weight for height (% of children under five; total, female, and male)	
	2.2.1 Prevalence of wasting, weight for height (% of children under five; total, female, and male)	
Water, sanitation, and hygiene (WASH)	3.9.2 Mortality rate attributed to unsafe water, unsafe sanitation, and lack of hygiene (exposure to unsafe water, sanitation, and hygiene for all (WASH) services)	No data variable
Energy	3.4.1 Mortality from cardiovascular disease, cancer, diabetes, or chronic respiratory disease between exact ages 30 and 70 (%)	As for population
	3.9.1 Mortality rate attributed to household and ambient air pollution	No data variable
Cities and human settlements: Climate change	3.3.3 Malaria incidence per 1000 population	Data not available
	11.5.1 Number of deaths, missing persons, and directly affected persons attributed to disasters per 100,000 population	No data variable

Sources: DataBank from the World Bank Group [18].
